# LRRC4 functions as a neuron-protective role in experimental autoimmune encephalomyelitis

**DOI:** 10.1186/s10020-021-00304-4

**Published:** 2021-05-01

**Authors:** Yan Zhang, Di Li, Qiuming Zeng, Jianbo Feng, Haijuan Fu, Zhaohui Luo, Bo Xiao, Huan Yang, Minghua Wu

**Affiliations:** 1 Hunan Provincial Tumor Hospital and the Affiliated Tumor Hospital of Xiangya Medical School, Central South University, Changsha, 410013 Hunan China; 2 The Key Laboratory of Carcinogenesis of the Chinese Ministry of Health, The Key Laboratory of Carcinogenesis and Cancer Invasion of the Chinese Ministry of Education, Cancer Research Institute, Central South University, Changsha, 410008 Hunan China; 3 Department of Pathology, Affiliated Hospital of Guilin Medical University, Guilin, 541001 Guangxi China; 4 Internal Medicine-Neurology, Xiangya Hospital, Central South University, Changsha, 410008 Hunan China

**Keywords:** LRRC4, NGL-2, Multiple sclerosis, NF-κB, Rab7b

## Abstract

**Background:**

Leucine rich repeat containing 4 (LRRC4), also known as netrin-G ligand-2 (NGL-2), belongs to the superfamily of LRR proteins and serves as a receptor for netrin-G2. LRRC4 regulates the formation of excitatory synapses and promotes axon differentiation. Mutations in LRRC4 occur in Autism Spectrum Disorder (ASD) and intellectual disability. Multiple sclerosis (MS) is a chronic neuroinflammatory disease with spinal cords demyelination and neurodegeneration. Here, we sought to investigate whether LRRC4 is involved in spinal cords neuron-associated diseases.

**Methods:**

LRRC4 was detected in the CNS of experimental autoimmune encephalomyelitis (EAE) mice by the use of real-time PCR and western blotting. LRRC4^−/−^ mice were created and immunized with myelin oligodendrocyte glycoprotein peptide (MOG)_35–55_. Pathological changes in spinal cords of LRRC4^−/−^ and WT mice 15 days after immunization were examined by using hematoxylin and eosin (H&E), Luxol Fast Blue (LFB) staining and immunohistochemistry. The number of Th1/Th2/Th17/Treg cells in spleens and blood were measured with flow cytometry. Differential gene expression in the spinal cords from WT and LRRC4^−/−^ mice was analyzed by using RNA sequencing (RNA-seq). Adeno-associated virus (AAV) vectors were used to overexpress LRRC4 (AAV-LRRC4) and were injected into EAE mice to assess the therapeutic effect of AAV-LRRC4 ectopic expression on EAE.

**Results:**

We report that LRRC4 is mainly expressed in neuron of spinal cords, and is decreased in the spinal cords of the EAE mice. Knockout of LRRC4 have a disease progression quickened and exacerbated with more severe myelin degeneration and infiltration of leukocytes into the spinal cords. We also first found that Rab7b is high expressed in EAE mice, and the deficiency of LRRC4 induces the elevated NF-κB p65 by up-regulating Rab7b, and up-regulation of IL-6, IFN-γ and down-regulation of TNF-α, results in more severe Th1 immune response in LRRC4^−/−^ mice. Ectopic expression of LRRC4 alleviates the clinical symptoms of EAE mice and protects the neurons from immune damages.

**Conclusions:**

We identified a neuroprotective role of LRRC4 in the progression of EAE, which may be used as a potential target for auxiliary support therapeutic treatment of MS.

**Supplementary Information:**

The online version contains supplementary material available at 10.1186/s10020-021-00304-4.

## Background

Leucine rich repeat containing 4 (LRRC4), also known as netrin-G ligand-2 (NGL-2), belongs to the superfamily of LRR proteins and serves as a ligand for netrin-G2 (Woo et al. 2009). LRRC4 regulates the formation of excitatory synapses by clustering excitatory postsynaptic proteins, participates in the differentiation of neurons, and promotes neurite extension specific dendritic segments of hippocampal neurons (Kim et al. 2006; Wu et al. 2007; DeNardo et al. 2012; Li et al. 2014). LRRC4 associates with the polarity-associated partitioning defective (PAR) complex through binding to PAR6 to stabilize axonal microtubules and promote axon differentiation via the aPKCζ/MARK2 pathway (Xu et al. 2015). Knockout of LRRC4 in mice suppresses NMDAR-dependent synaptic plasticity, and display mild autistic-like behaviors (Um et al. 2018). LRRC4 also acts as a tumor suppressor gene and significantly inhibits glioma cell proliferation by regulating RTK/ERK/AKT, SDF-1α/CXCR4 and cytokines such as VEGF and TGF-β to inhibit glioblastoma cell proliferation, migration and angiogenesis (Wu et al. 2006, 2008a, b). It has been recently shown that LRRC4 binds to phosphoinositide-dependent protein kinase 1 (PDPK1), facilitates activation of NF-κB of GBM cells, and promotes the secretion of IL-6, CCL2 and IFN-γ, and inhibits tumor-infiltrating Treg cell expansion and GBM cell growth (Li et al. 2017). Moreover, mutations of LRRC4 gene in humans have been implicated in Autism Spectrum Disorder (ASD), and intellectual disability (Jiang et al. 2013; Sangu et al. 2017). Although locomotor training increases synaptic structure with high NGL-2 expression after spinal cord hemisection (Kobayakawa et al. 2019), the effect of LRRC4 on spinal cords-associated diseases hasn’t known.

Multiple sclerosis (MS) is a chronic neurodegeneration disease of the central nervous system (CNS) that often presents in young adults (International Multiple Sclerosis Genetics 2019). The pathological mechanism of MS is not completely clear. The genomic map implicates peripheral immune cells and microglia in susceptibility (Steinman 2014). The dysregulation of the immune system is widely considered to be the factor for both initiation and progression of MS, in which CD4^+^ T-helper (Th) cells play an important role (Riedhammer et al. 2015; Segal 2019). Th1 and Th17 cells play complementary roles in the pathogenesis of experimental autoimmune encephalomyelitis (EAE). Th1 cells produce cytokines, such as interferon (IFN)-γ, interleukin (IL)-2 and tumor necrosis factor (TNF)-β, which can induce cell-mediated immunity and phagocyte-dependent inflammation (Hu et al. 2016). Th17 cells have been defined as a distinct subset of CD4^+^ T cells that produce IL-17A, IL-17F, IL-21, IL-22 and TNF-α, promote inflammation, and are pathogenic in many autoimmune disorders (Fletcher et al. 2010). In contrast to Th1 and Th17 cells, Th2 cells secrete IL-4, -10, and -13 and induce strong antibody responses while inhibiting several functions of phagocytic cells (Codarri et al. 2011). Regulatory T (Treg) cells are thought to play a central role in the maintenance of peripheral immune tolerance (Kleinewietfeld et al. 2014). Treg cells protect the mice from developing chronic EAE, implying that Treg cells contribute to the protection of individuals against MS (Nie et al. 2015). Thus, the balance among Th1, Th17 and Treg cells appears critical in MS pathogenesis, and regulation of Th cell differentiation may prove to be a potential strategy for MS diagnosis and treatment (Kleinewietfeld et al. 2013).

In this study, by using a murine model of EAE, which is commonly employed as a model for MS, we discovered that LRRC4 is mainly expressed in neuron of spinal cords, the level of LRRC4 decreases in the spinal cords of the EAE mice. Deletion of LRRC4 accelerates demyelination of spinal cords and infiltration of leukocytes, exacerbates the neurological function damage, promote disease progression of EAE.

## Methods

### Mice and EAE induction

C57BL/6 mice were purchased from the Slake Experimental Animal Company. LRRC4-loxP chimeric mice were constructed by Cyagen Biosciences, crossbred with widespread expression cre mice [B6.C-Tg (CMV-cre) 1Cgn/J, Jackson Laboratory], and then hybridized offspring of LRRC4^−/−^ mice were obtained. The genotypes of mice were identified with real-time PCR and western blotting. Wild-type littermate controls with same genetic background were used. All mice were maintained in specific pathogen-free conditions in the Laboratory Animal Department of Central South University. The mouse protocols were performed in accordance with the guidelines for the care of laboratory animals and the Animal Care and Use Committee of Central South University. EAE was induced by the use of myelin oligodendrocyte glycoprotein (MOG_35–55_, H-MEVGWYRSPFSRVVHLYRNGK-OH, GL Biochem) in 8-week-old female mice. Each mouse was immunized by subcutaneously injection with 200 μg of MOG_35–55_ emulsified in Complete Freund’s Adjuvant (CFA, Sigma) with 500 μg mycobacterium tuberculosis (strain H37RA, DIFCO). Mice were then intraperitoneally injected with 200 ng of pertussis toxin (List Biological Laboratories) at the time of immunization and 2 days after. Mice were evaluated for clinical scoring and body weight daily after immunization for 30 days. Neurological signs were recorded to evaluate motor deficit as follows: 0, no deficit; 1, dysfunction of tail; 2, dysfunction of tail and one limb; 3, limp tail and dysfunction of two limbs; 4, disturbed function in tail and all limbs; 5, moribund state or death. Scoring was performed in a blinded fashion. The study was approved by the Joint Ethics Committee of the Central South University Health Authority.

### Neuronal culture and electrotransfection

Newborn mice were sacrificed by decapitation and sterilized by using 70% ethanol. Hippocampus was isolated and digested with 0.25% trypsin–EDTA (HyClone) for 30 min at 37 °C, followed by trituration with pipetting in DMEM-F12 (HyClone) medium. Dissociated neurons were transfected using electrotransfection. Neurons were plated onto dishes coated with poly-d-lysine (0.1 mg/ml, Sigma). After cultivation for 4 h, the media were replaced with neuronal culture medium (Gibco) containing 1% glutamate (Sigma) and 2% B27 medium (Sigma) at 37 °C and 5% CO_2_ in a humidified atmosphere.

### RNA interference and adeno-associated virus infection

The target sequences of Rab7b shRNA1 and shRNA2 were 5′-AGTGGACTTGAAACTTATCATTGTTGGTG-3′ and 5′-AAGTTAGTGCGAAGAATGACATCAATGTG-3′, respectively. All the DNA segments were synthesized by Sangon Biotech and inserted into the pSuper vector. LRRC4 and Rab7b were amplified from mouse brains and cloned into pcDNA3.1 plasmid. Transfection of plasmids was conducted by following the manufacturer’s instructions. The adeno-associated virus (AAV) vector for overexpressing LRRC4 (AAV-LRRC4) and controls (AAV-CON) were constructed by Vigene Biosciences. AAV-LRRC4 was packaged in HEK293T cells, and the cells were then lysed by the use of freeze–thaw cycles. The viruses were purified using the iodixanol gradient ultracentrifugation method. The viruses for AAV-LRRC4 or AAV-CON were injected intravenously at the tail at a dose of 5 × 10^12^ vector genome (vg)/kg 7 days before immunization. All these AAV-viral used in this study were biosafety and the procedures were performed using a protocol approved by the Joint Ethics Committee of the Central South University Health Authority.

### Quantitative reverse transcription-PCR (qRT-PCR)

Total RNA was extracted from tissues or cultured cells by using the TRI reagent (Molecular Research Center, MRC) according to the manufacturer’s instructions. 2 μg total RNA was reverse transcribed to cDNA using the RevertAid First Strand cDNA Synthesis Kit (Thermo Fisher) according to the manufacturer’s instructions. Real-time PCR analyses were performed using with SYBR Green PCR kits (Bimake) following manufacturer’s instructions. The primers used were as described in Table 1.

**Table 1:** A list of primers used in real-time PCR analysis

Gene	Forward	Reverse
GAPDH	AGTGGCAAAGTGGAGATTGTTG	TGTTAGTGGGGTCTCGCTCC
LRRC4	TGCTGCCATGTTGATTGTC	GTGCTGGTTTGTAGGTGTTGT
Rab7b	TGGCAAAGATTATCCCTA	ATTCTTCGCACTAACTTCA
IL-6	TTGCCTTCTTGGGACTGATG	CACGATTTCCCAGAGAACATG
IL-10	ACATACTGCTAACCGACTCC	AGGGTCTTCAGCTTCTCAC
TNF-α	GGCGGTGCCTATGTCTCAG	GGCTACAGGCTTGTCACTCG
IL-17A	CTCAGACTACCTCAACCGTTC	TGTGGTGGTCCAGCTTTCC
TGF-β	AGCAACAATTCCTGGCGATAC	CTAAGGCGAAAGCCCTCAAT
IFN-γ	CAAGTGGCATAGATGTGGAAG	TGCTGATGGCCTGATTGTC

### Western blotting

Western blotting analysis was conducted according to standard procedures. Tissues or cultured cells were collected and lysed in lysis buffer (300 mM NaCl, 50 mM Tris pH 8.0, 0.4% NP-40, 10 mM MgCl_2_, and 2.5 mM CaCl_2_) supplemented with protease inhibitors cocktail (Bimake) and phosphatase inhibitors (Bimake). 30 μg protein samples were loaded onto 10% sodium dodecyl sulfate–polyacrylamide gel electrophoresis (SDS-PAGE), transferred to polyvinylidene fluoride (PVDF, Millipore) membrane, blocked by 5% non-fat milk, and incubated with different primary antibodies and secondary antibodies. Signals were detected using chemiluminescent HRP substrate (Millipore). The primary antibodies used were as follows: LRRC4, Rab7b (Abcam), NF-κB p65, ERK1/2, p-ERK1/2 (Thr202/Tyr204), p-AKT (Ser473) (Cell Signaling Technology), AKT and GAPDH (Proteintech).

### Immunohistochemistry

Mice were anesthetized using barbital sodium and then intracardially perfused with normal saline and 4% paraformaldehyde for fixation. Spinal cords were dissected, fixed in 4% paraformaldehyde at 4℃ overnight, and sliced in 4 μm while pathological changes were examined with hematoxylin and eosin (H&E) staining and Luxol Fast Blue (LFB) staining. For immunohistochemistry analysis, sections were blocked with 3% hydrogen peroxide for 10 min and normal goat serum for 1 h at room temperature. The sections were then incubated at 4℃ overnight with anti-GFAP (Abcam), IBA1 (Thermo Scientific) antibodies, biotinylated secondary antibody (Maxim Biotechnologies) for 20 min followed by the treatment of streptavidin-conjugated HRP (Maxim Biotechnologies) for 10 min. Detection was enabled with 3,3-diaminobenzidine (DAB; Maxim Biotechnologies) treatment, while hematoxylin was used for counterstaining. The cell number of infiltrated lymphocytes or microglia or astrocytes in spinal cords were counted by Image-pro Plus software.

### Immunofluorescence

The mice brains were separated, fixed in 4% paraformaldehyde at 4℃ overnight, and embed with OCT compound and the frozen tissues were sliced in 4 μm. For immunofluorescence analysis, sections were blocked with normal goat serum for 1 h at room temperature. Then, the sections were incubated with anti-GFAP, IBA1, TAU (Abcam) and LRRC4 antibodies at 4℃ overnight, and FITC-conjugated goat anti-rabbit, Cy3-conjugated goat anti-moused secondary antibody (Proteintech) for 1 h at room temperature, and DAPI staining for 5 min. The pictures were captured by using olympus fluorescence microscope.

### Flow cytometry

Spleens and blood from the mice were harvested, and a single cell suspension was prepared. For quantification of the number of Th1/Th2/Th17 cells, cells were stimulated with PMA (50 ng/ml, Sigma), activation cocktail (750 ng/ml, BioLegend), and monensin (2 μmol/L, BioLegend). Cells were thereafter stained with FITC-conjugated anti-CD4 antibodies (BioLegend), permeabilized with permeabilization solution, and then stained with PE/Cy7-conjugated anti-IFN-γ, PE-conjugated anti-IL-4 and APC-conjugated anti-IL-17A (BioLegend). For detection of Treg cells, the cells were stained with PE-conjugated anti-CD25 and Alexa Fluor 647-conjugated anti-Foxp3 (BioLegend). Fluorescence was examined with FACS Canto II (BD Biosciences). All flow cytometry data presented herein were gated by the use of CD4^+^, while IFN-γ^+^ cells represented Th1 cells, IL-4^+^ cells represented Th2 cells, IL-17A^+^ cells represented Th17 cells, and CD25^+^Foxp3^+^ cells represented Treg cells. Data were analyzed with the FlowJo software.

### RNA sequencing and bioinformatic analyses

The spinal cords of thoracic vertebra were isolated from WT and LRRC4-KO 4-weeks female mice. We generated RNA sequencing libraries from these spinal cords using the Illumina TruSeq RNA sample kits, according to the manufacturer’s instructions. Libraries were validated using the Agilent Technologies 2100 Bioanalyzer and Quant-iT™ dsDNA HS Assay (Life Technologies). We quantified gene expression as FPKM (Fragments Per Kilobase of gene model per Million reads per sample) using Cufflinks. We identified differentially expressed genes from RNA-Seq data using SAM analysis at FDR < 0.01%. We generated rank order gene lists by ordering expressed genes by their FPKM value and comparing the relative ranking of the gene between WT and LRRC4-KO mice.

### Statistical analysis

All the experiments were repeated at least three times, and the representative data are shown. The statistical analysis was performed using GraphPad Prism 5 and SPSS version 17.0. Data analysis was performed with Student’s t test and one-way ANOVA and presented as the mean ± SEM. P values less than 0.05 were considered significant.

## Results

### Down-regulation of LRRC4 in neuron of spinal cord during EAE pathogenesis

**Fig. 1:**
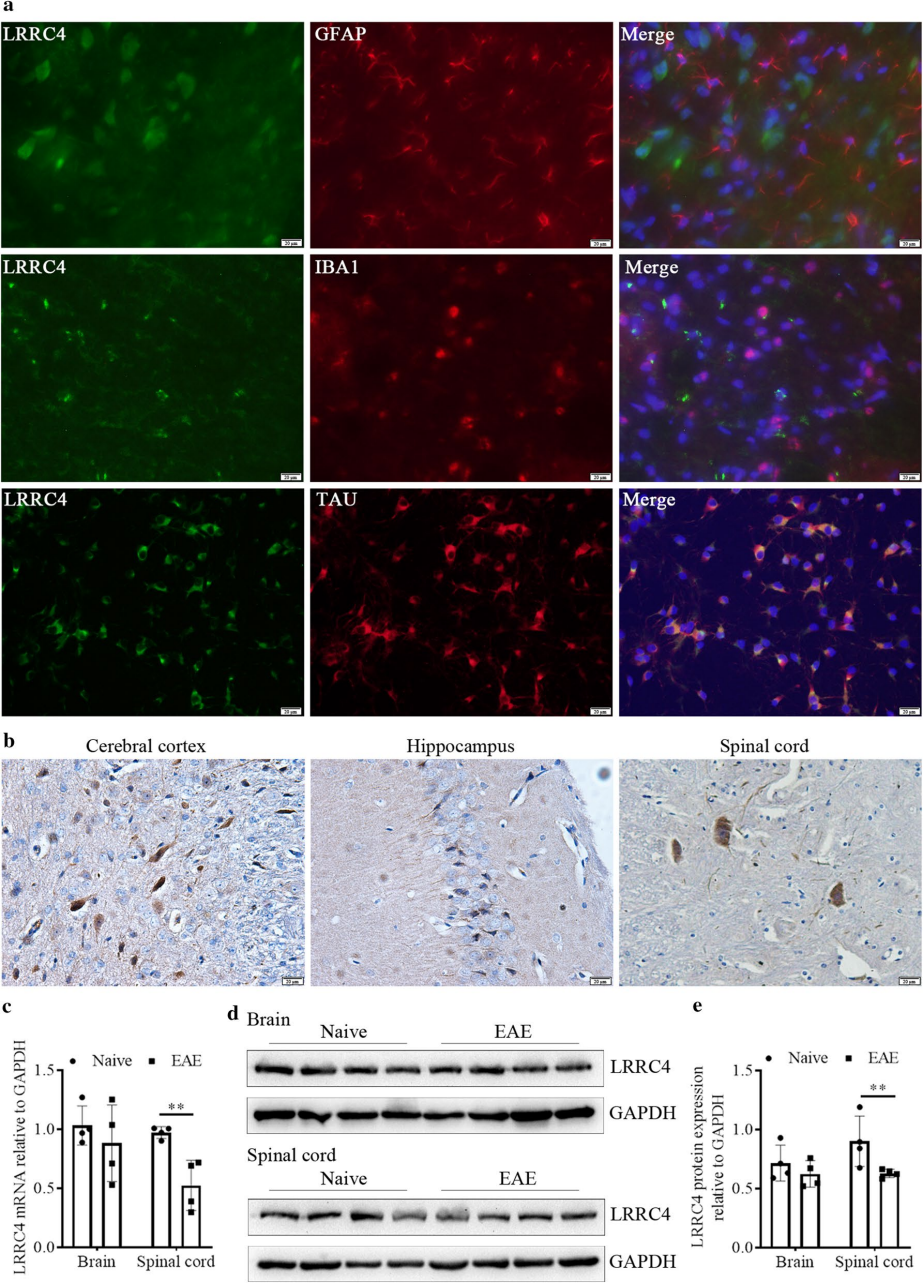
LRRC4 is down-regulated in spinal cords during EAE. **a** Immunofluorescence staining analysis of LRRC4 expression co-localization with GFAP, IBA1 and TAU. **b** Immunohistochemistry staining analysis of LRRC4 expression in mouse brains and spinal cords. **c** Real-time PCR analysis of LRRC4 mRNA levels in the brains and spinal cords of healthy (naïve) and EAE mice. All values are normalized to the level (= 1) of one of healthy mice. Results are shown as means ± SEM (n = 4). **d** Western blotting analysis of LRRC4 protein levels in the brains and spinal cords of healthy and EAE mice. **e** Quantification of the western blotting images in **d**. All values are represented as the values of LRRC4 protein levels divided by those of GAPDH. Results are shown as means ± SEM (n = 4). **p < 0.01

Firstly, we detected the expression of LRRC4 in neurons, astrocytes and microglia in normal mice brains and neurons by immunofluorescence staining with anti-LRRC4 and GFAP (astrocytes marker), IBA1 (microglia marker) and TAU (neurons marker) antibody. We found that LRRC4 expressed in neurons but not in astrocytes or microglia (Fig. 1a). Immunohistochemistry staining was also certified these results that LRRC4 expressed in neurons whatever it was in the cerebral cortex, hippocampus, or spinal cord (Fig. 1b). EAE mice were induced by MOG_35–55_, the clinical scoring was recorded daily, and the EAE mice began to appear neurological sign (dysfunction of tail) on the seventh day (Additional file 1: Fig. S1a). H&E and LFB staining revealed increased lymphocyte infiltration and demyelinated lesion in EAE mice (Additional file 1: Fig. S1b). In EAE mice, the expression of LRRC4 was detected at before or 15 days after treatment. Relative to the healthy mice, both LRRC4 mRNA and protein expression were significantly reduced in the spinal cords of EAE mice, while no difference was detected in the brains (Fig. 1c–e), suggesting that MOG_35–55_ induced LRRC4 downregulation in neuron of spinal cords, and was involved in the progression of EAE mice.

### LRRC4 functions as a neuron-protective role in EAE development

**Fig. 2:**
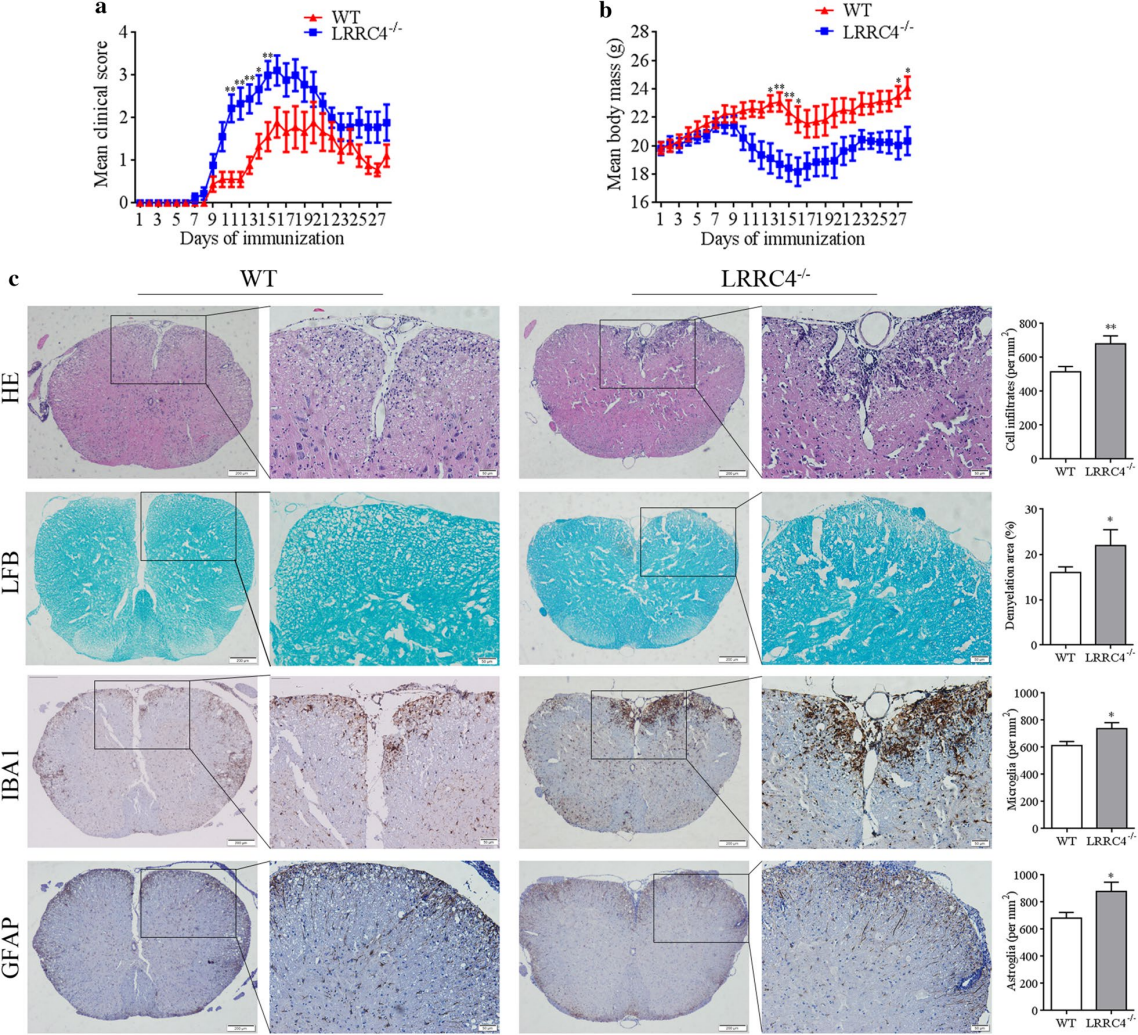
LRRC4 deletion leads to exacerbated EAE progression. **a** Clinical scores of MOG-immunized WT and LRRC4^−/−^ mice. Data represent the mean clinical scores of 10 mice per group ± SEM. **b** Body mass of WT and LRRC4^−/−^ mice. Data represent the mean body mass of 10 mice per group ± SEM. **c** Histopathological analysis of neuroinflammation and demyelination in spinal cords of WT and LRRC4^−/−^ mice 15 days after EAE induction. Sections were stained by the use of H&E staining, LFP staining and immumohistochemical staining with anti-IBA1 and -GFAP antibody. *p < 0.05, **p < 0.01

Thinking that MOG_35–55_ induced EAE due to demyelination, therefore, we proposed whether LRRC4 function as neuroprotective effect in spinal cords during EAE process. We constructed mice with LRRC4 deletion (LRRC4^−/−^) and subsequently induced EAE in LRRC4^−/−^ mice and the wild type (WT) littermates (Additional file 1: Fig. S2a–c). Consistent with the earlier report by Zhang et al., LRRC4^−/−^ mice showed decreased threshold of auditory brainstem response (ABR) (Additional file 1: Fig. S2d, e), LRRC4^−/−^ mice were reduced in synchronization of auditory neurons in the spiral ganglia (Zhang et al. 2008). In EAE model, the neurological function score indirectly reflects the damage of neurons of mice spinal cord. LRRC4^−/−^ mice exhibited much higher neurological function score (Fig. 2a) and accelerated loss of body mass (Fig. 2b) than WT mice with the difference peaking at day 16. At the same time, we used LFB staining of the spinal cords collected at day 15 after MOG_35–55_ treatment and found more severe demyelination in LRRC4^−/−^ mice than in WT mice (Fig. 2c). In addditon, H&E staining and immunohistochemistry analysis with anti-IBA1 and GFAP antibodies revealed increased lymphocyte infiltration and increased density of microglia and astrocytes around demyelinated lesion sites in LRRC4^−/−^ mice compared with WT mice. The aboved results indicated that LRRC4 funcions as a neuron-protective role in EAE disease development. The loss of LRRC4 leads to the aggravated demyelination of spinal cord and accelerated recruitment of lymphocytes to demyelinated lesion.

### LRRC4 deletion up-regulates NF-κB in EAE mice

**Fig. 3:**
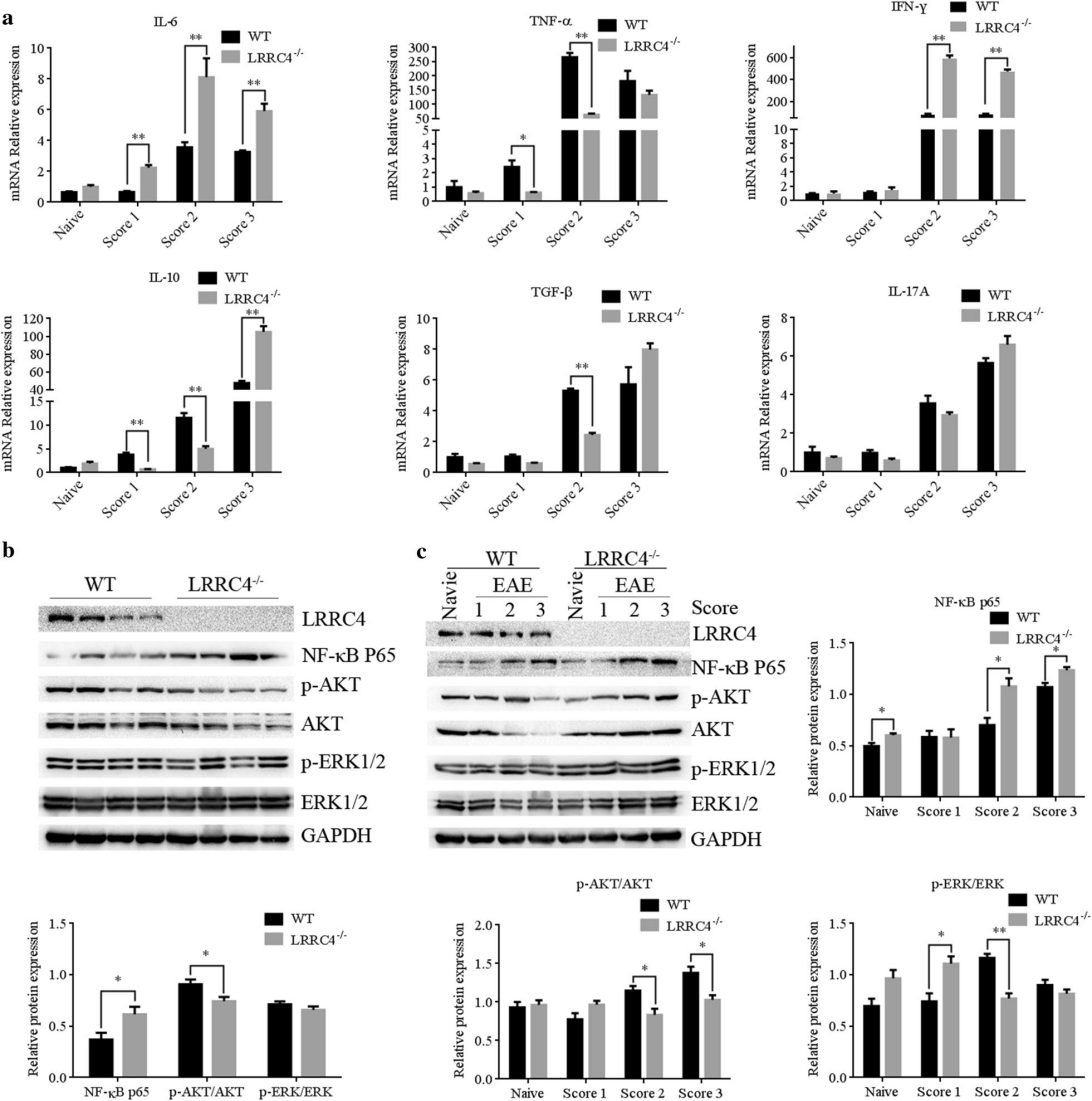
LRRC4 deletion up-regulates NF-κB in EAE mice. **a** Real-time PCR analysis of cytokine mRNA levels of spinal cords in WT and LRRC4^−/−^ mice that are healthy or with different clinical scores. **b** Western blotting analysis and quantification of the western blotting images of spinal cord lysates from WT and LRRC4^−/−^ mice. Each lane represents samples from an individual mouse. **c** Western blotting analysis and quantification of the western blotting images of spinal cord lysates in WT and LRRC4^−/−^ mice that are healthy or with different clinical scores. Results are shown as means ± SEM (n = 3). *p < 0.05, **p < 0.01

Given the higher concentration of lymphocytes in the CNS of LRRC4^−/−^ mice upon the induction of EAE, we hypothesized that LRRC4^−/−^mice had higher pro-inflammatory and lower anti-inflammatory cytokines in their spinal cords than did wild-type mice in EAE development. So we examined the levels of various cytokines. We found that the levels of IL-6 mRNA were consistently elevated in the spinal cords of LRRC4^−/−^ mice with different scores of EAE when compared with WT mice (Fig. 3a). In addition, IFN-γ mRNA levels were enhanced at score 2 and 3 of EAE in spinal cords of LRRC4^−/−^ mice. In contrast, IL-10 mRNA levels were reduced at the score 1 and 2 of EAE in spinal cords of LRRC4^−/−^ mice, while increasing at the score 3 in LRRC4^−/−^ mice. Meanwhile, TNF-α mRNA levels were reduced at the score 1 and 2 in spinal cords, while TGF-β levels decreased at the score 2 of spinal cords in LRRC4^−/−^ mice. In contrast, IL-17A mRNA levels exhibited no significant difference in spinal cords between LRRC4^−/−^ and WT mice.

After showing that LRRC4 deletion caused elevated inflammatory responses in the spinal cords and exacerbation of EAE pathogenesis, we sought to dissect the underlying molecular mechanisms. LRRC4 has been reported to inhibit NF-κB activation and regulate the ERK/MAPK and PI3K/AKT pathway (Wu et al. 2006). NF-κB plays an important role in controlling expression of genes including pro-inflammatory cytokines, chemokines, nitric oxide synthases and cell adhesion molecules related to the pathogenesis of autoimmunity (Yan et al. 2008). The ERK/MAPK and PI3K/AKT signaling pathways reportedly modulate Tregs and Th17 cells differentiation, while the PI3K/AKT signaling pathway promotes oligodendrocyte differentiation and myelination (Hou et al. 2018). The well established role of NF-κB, ERK/MAPK and PI3K/AKT signaling in inflammation and EAE pathogenesis led us to investigate whether they might play a role in mediating the function of LRRC4 in EAE.

We first assessed the levels of NF-κB, p-AKT and p-ERK1/2 in LRRC4^−/−^ and WT mice under naïve and EAE conditions. Under naïve conditions, NF-κB p65 was up-regulated, while the ratio of p-AKT/AKT was reduced in the spinal cords of LRRC4^−/−^ mice compared with that of WT mice (Fig. 3b). After immunization, LRRC4^−/−^ mice expressed higher levels of NF-κB p65 than WT mice, at clinical score 2 and 3 in the spinal cords (Fig. 3c). The ratio of p-AKT/AKT decreased in spinal cords of LRRC4^−/−^ mice at score 2 and 3 compared with that of WT mice. In contrast, the ratio of p-ERK/ERK exhibited little difference in spinal cords between WT and LRRC4^−/−^ mice, while increasing at score 2 and decreasing at score 3 in spinal cords of LRRC4^−/−^ mice. Thus, LRRC4 deletion induces an up-regulation of NF-κB, causing alterations in the levels of inflammation-related cytokines in the spinal cords, which may contribute to accelerated progression of EAE.

### Rab7b is correlated inversely with LRRC4 in EAE

**Fig. 4:**
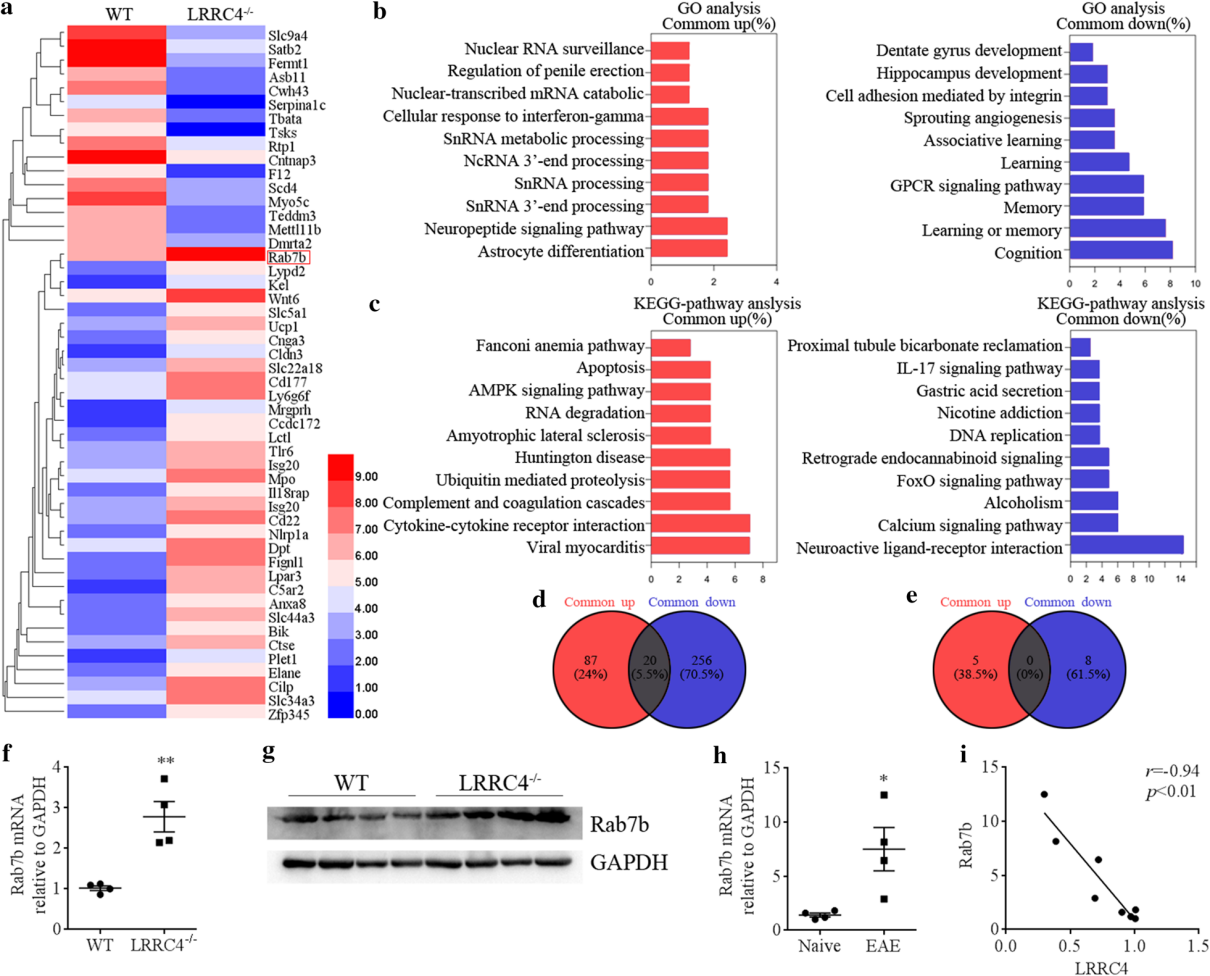
RNA-seq analysis revealed a role of Rab7b in EAE. **a** A heatmap showing DEGs in spinal cord of WT mice versus LRRC4^−/−^ mice from cluster analysis of RNA-seq. **b** The top 10 GO terms of up-regulated and down-regulated DEGs between WT and LRRC4^−/−^ mice. **c** The top 10 KEGG pathways of up-regulated and down-regulated DEGs between WT and LRRC4^−/−^ mice. **d** The comparison of GO terms between up-regulated DEGs and down-regulated DEGs. **e** Comparison of KEGG pathways between up-regulated DEGs and down-regulated DEGs. **f** Rab7b mRNA levels of spinal cords in WT and LRRC4^−/−^ mice as determined with real-time PCR. Results are shown as means ± SEM (n = 4). **g** Rab7b protein levels of spinal cords in WT and LRRC4^−/−^ mice as assessed with western blotting. **h** Rab7b mRNA levels of the spinal cords in healthy and EAE mice assessed with real-time PCR. Results are shown as means ± SEM (n = 4). **i** Correlation analysis of Rab7b and LRRC4 mRNA levels in healthy and EAE mice. *p < 0.05, **p < *0.01*

To obtain a more comprehensive understanding of the distinct molecular programs between WT mice and LRRC4^−/−^ mice, we isolated the spinal cords from WT and LRRC4^−/−^ mice before or 15 days after immunization and analyzed them by using RNA sequencing (RNA-seq) (Fig. 4 and Additional file 1: Fig. S3). The top 50 genes in WT versus LRRC4^−/−^ mice were selected for cluster analysis (Fig. 4a). We performed GO and KEGG analyses to investigate the molecular function and biological pathways of the differentially expressed genes (DEGs). The top 10 enriched GO terms and KEGG pathways of the up-regulated and down-regulated DEGs, according to the percentage of genes, were selected (Fig. 4b, c). For instance, Fig. 4b showed that cellular response to IFN-γ was among the enriched GO terms of up-regulated DEGs, consistent with earlier findings that IFN-γ^−/−^and IFN-γR^−/−^ mice show more severe and chronic-progressive course of EAE (Sosa et al. 2015). In Fig. 4c, we are surprised finding that the KEGG pathway of up-regulated DEGs by LRRC4 decency is associated with amyotrophic lateral sclerosis, in which Rab7b was obvious upregulated gene and of special interest, and it was recently shown that Rab7b is involved in activation of NF-κB and enhances the production of IL-6 (He et al. 2011). Moreover, we also compared common DEGs between the up-regulated and down-regulated DEGs (Fig. 4d, e), the number of shared DEGs in GO terms between up-regulated and down-regulated DEG was small.

Next, we examined Rab7b expression in the spinal cords of WT and LRRC4^−/−^mice and found that both the mRNA and protein levels were elevated in LRRC4^−/−^ mice compared with WT mice (Fig. 4f, g). We also assessed Rab7b mRNA expression in the spinal cords of naïve mice and EAE mice and detected increased Rab7b mRNA expression in the spinal cords of EAE mice (Fig. 4h). Furthermore, Rab7b mRNA levels correlated inversely with LRRC4 expression (Fig. 4i). Thus, Rab7b expression in the spinal cords of EAE mice is elevated upon LRRC4 deletion, raising the possibility that Rab7b may be involved in EAE pathogenesis.

### Rab7b mediates NF-κB up-regulation in LRRC4^−/−^ neurons

**Fig. 5:**
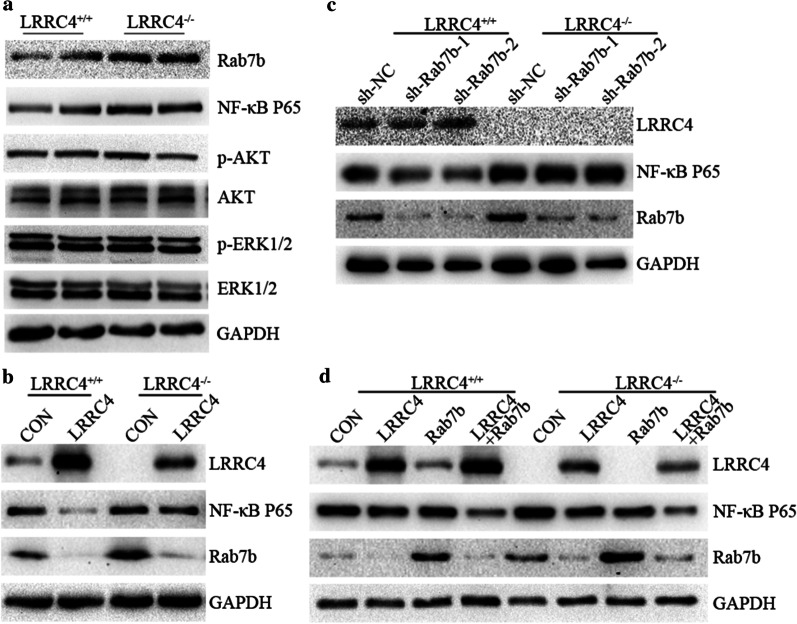
Rab7b mediates NF-κB up-regulation in LRRC4^−/−^ Neurons. **a** Western blotting analysis of various proteins in LRRC4^+/+^ and LRRC4^−/−^ neurons. **b** Western blotting analysis of various proteins in LRRC4^+/+^ and LRRC4^−/−^ neurons after ectopic expression of LRRC4. **c** Western blotting analysis of various proteins in LRRC4^+/+^ and LRRC4^−/−^ neurons after knockdown of Rab7b. **d** Western blotting analysis of various proteins in LRRC4^+/+^ and LRRC4^−/−^ neurons co-transfected with Rab7b and LRRC4

The elevated Rab7b expression in LRRC4^−/−^ mice susceptible to EAE led us to determine the molecular link between LRRC4, Rab7b and NF-κB. To do so, we isolated mouse neurons of WT and LRRC4^−/−^ mice and subsequently determined Rab7b and NF-κB p65 expression. As expected, Rab7b and NF-κB p65 were up-regulated in LRRC4^−/−^ neurons compared with LRRC4^+/+^ neurons (Fig. 5a). Ectopic expression of LRRC4 with the pcDNA3.1-LRRC4 expression vector induced a reduction of Rab7b and NF-κB p65 expression (Fig. 5b). RNAi-mediated knockdown of Rab7b in LRRC4^+/+^ neurons caused NF-κB p65 expression to reduce but not in LRRC4^−/−^ neurons (Fig. 5c). Ectopic expression of Rab7b had little effect on NF-κB p65 expression but strongly inhibited NF-κB p65 expression when Rab7b was co-transfected with LRRC4 (Fig. 5d). Thus, Rab7b regulates NF-κB in the presence of LRRC4 but plays no such role in LRRC4-deficient neurons. These results suggest that Rab7b might serve as a downstream effector of LRRC4 in the regulation of NF-κB.

### LRRC4 deletion developed more severe Th1 immune response

**Fig. 6:**
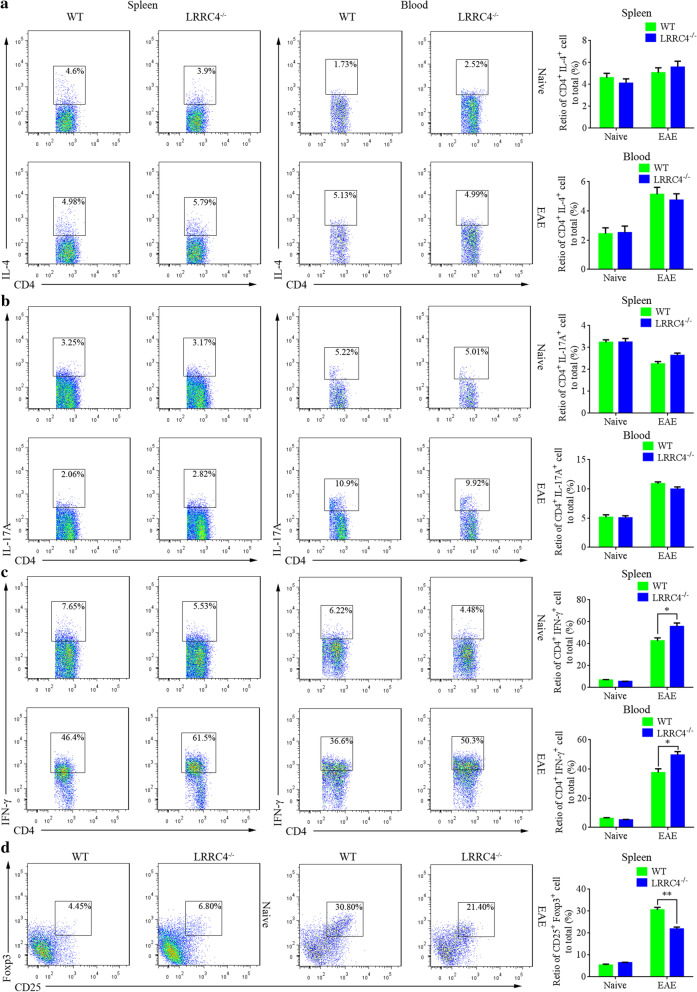
LRRC4 deletion developed more severe Th1 immune response. **a** Flow cytometry of Th2 (CD4^+^ IL-4^+^) cells of WT and LRRC4^−/−^ mice before or 15 days after EAE induction in spleens (left) or blood (right). **b** Flow cytometry of Th17 (CD4^+^ IL-17A^+^) cells of WT and LRRC4^−/−^ mice before or 15 days after EAE induction in spleens (left) or blood (right). **c** Flow cytometry of Th1 (CD4^+^ IFN-γ^ +^) cells of WT and LRRC4^−/−^ mice before or 15 days after EAE induction in spleens (left) or blood (right). **d** Flow cytometry of Treg (CD4^+^ CD25^+^ Foxp3^+^) cells of WT and LRRC4^−/−^ mice before or 15 days after EAE induction in spleens. *p < 0.05, **p < 0.01

Our previous study revealed that in GBM cells, LRRC4 interacted with phosphoinositide-dependent protein kinase 1 (PDPK1) and HSP90, which lead to IKKβ phosphorylation and NF-κB activation, and IL-6, CCL2, and IFN-γ production. IL-6 induced the naïve T cells differentiated into Teff cells but not Treg cells. Moreover, LRRC4 inhibited PDL-1 packing into exosomes and attenuated the interaction of PDL-1 with PD-1 on TIL cells, resulted in reduced effects of TIL cells on GBM cells. In EAE mice, the degenerated myelin induced by MOG_35–55_ would be phagocytosed by activated macrophages and microglia, leading to antigen presentation in the CNS and enhanced myelin-specific autoimmune responses. LRRC4^−/−^ mice developed more severe myelin degeneration, so we speculated that LRRC4^−/−^ mice may cause a more severe immune response. We therefore examined whether LRRC4 deletion causes any alterations in helper T cells populations during EAE development. To do so, we measured the proportion of cytokine-producing cells in the spleen and blood from LRRC4^−/−^ and WT mice (both naïve mice and mice with EAE induction) 15 days after immunization by using flow cytometry. Intracellular staining of IL-4, IL-17A and IFN-γ showed that LRRC4 deletion did not change the proportion of Th2 (CD4^+^ IL-4^+^) cells or Th17 (CD4^+^ IL-17A^+^) cells in the spleen and blood (Fig. 6a, b) whether or not EAE occur. However, although LRRC4 deletion failed to change the proportion of Th1 (CD4^+^ IFN-γ^+^) cells in the spleen and blood of naïve mice, it increased the proportion of Th1 cells in the spleen and blood of EAE mice (Fig. 6c). We next assessed the effect of LRRC4 deletion on regulatory T (Treg) cells, which reportedly play a critical role in the regulation of immune processes during EAE. Little difference in Treg cells (CD4^+^CD25^+^FoxP3^+^) was found between naïve LRRC4^−/−^ and WT mice in the spleen. However, a marked reduction in Treg cells was seen in the spleen of LRRC4^−/−^ EAE mice compared with WT EAE mice (Fig. 6d). Thus, LRRC4 deletion caused a Th1 immune response and reduced immune modulation, which may contribute to EAE progression.

### Ectopic LRRC4 expression alleviates EAE progression

**Fig. 7:**
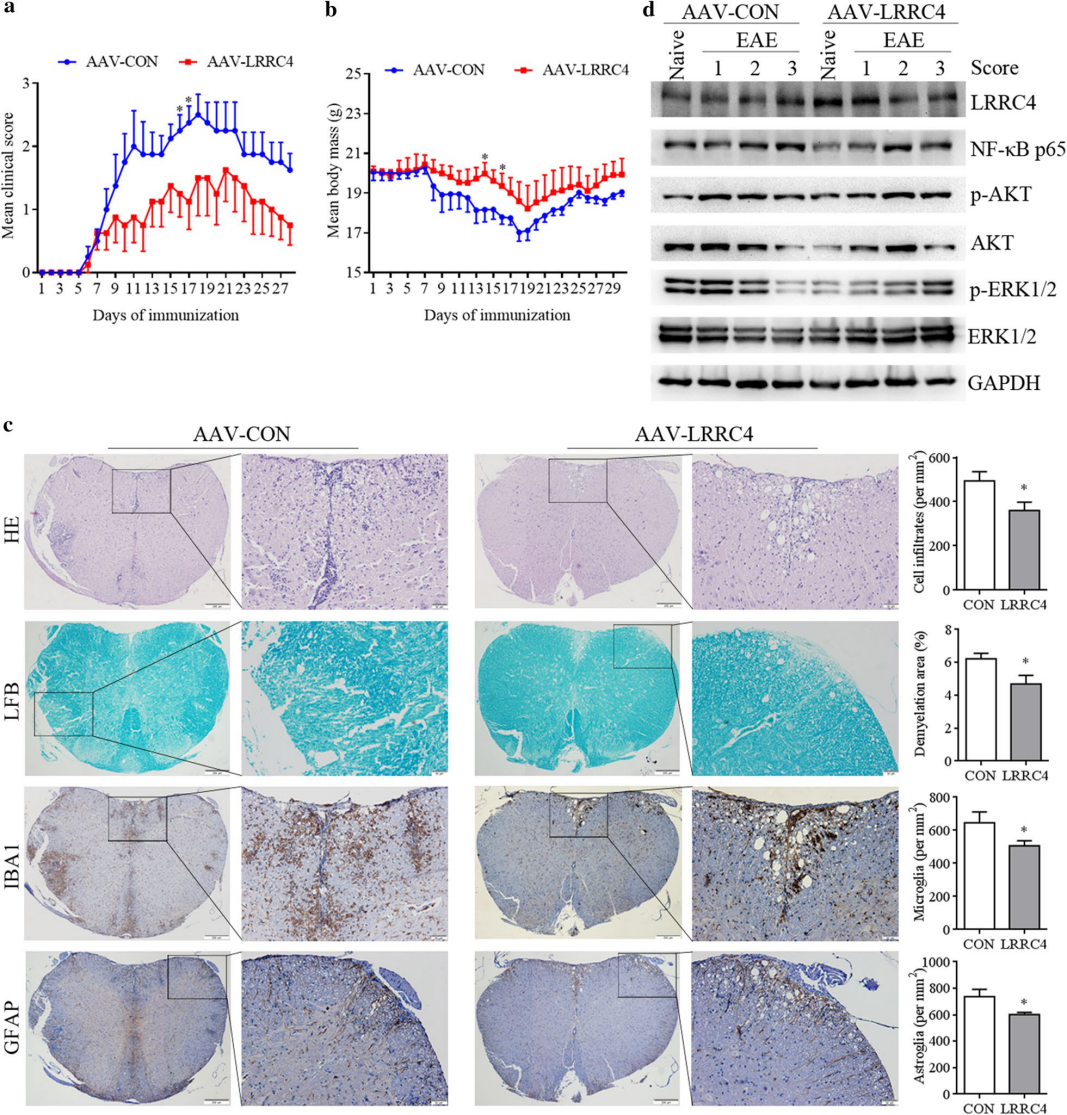
Ectopic LRRC4 expression alleviates EAE progression. **a** Clinical scores of MOG-immunized WT mice after intravenously injected with adeno-associated virus (AAV) vector containing LRRC4 (AAV-LRRC4) or control virus vector (AAV-CON). Data represent the mean clinical scores of 8 mice per group ± SEM. **b** Body mass of EAE mice after intravenously injected with AAV-LRRC4 and AAV-CON. Data represent the mean body mass of 8 mice per group ± SEM. **c** Histopathological analysis of neuroinflammation and demyelination in spinal cords of mice 15 days after EAE induction. Sections were subjected to H&E staining, LFP staining and immumohistochemical staining with anti-IBA1 and anti-GFAP antibody. **d** Western blotting analysis of various proteins in brains and spinal cords in EAE mice after intravenously injected AAV-LRRC4 and AAV-CON. *p < 0.05

Having shown that LRRC4 is down-regulated in EAE mice and that LRRC4 deletion leads to aggravated EAE progression, we asked whether ectopic LRRC4 expression could rescue the pathological defects of EAE. We injected adeno-associated virus (AAV) vector intravenously to ectopically express LRRC4 (AAV-LRRC4) or control virus vector (AAV-CON) in the mice and subsequently induced EAE with MOG_35–55_ 10 days after injection. Clinical scores of EAE were measured and documented daily, showing that AAV-LRRC4 injection alleviated the progression of EAE and body mass loss compared with AAV-CON injection (Fig. 7a, b). At the tissue level, AAV-LRRC4 injection caused lymphocyte infiltration into spinal cords to decrease in EAE mice, as revealed by H&E staining (Fig. 7c). In addition, experiments with luxol fast blue (LFB) staining showed that overexpression of LRRC4 decreased demyelination in the spinal cords of EAE mice, while the density of microglia and astrocytes were also reduced around demyelinated lesion sites in the spinal cords of mice injected with AAV-LRRC4, as illustrated by immunohistochemical analysis using anti-IBA1 and GFAP antibody (Fig. 7c). Thus, LRRC4 ectopic expression alleviates the defects in demyelination and autoimmunity caused by EAE. As such, AAV-LRRC4 virus may be potentially used as a therapeutic tool for treating MS patients.

Consistent with our earlier findings, NF-κB p65 was down-regulated in the spinal cords of mice injected with AAV-LRRC4. In contrast, the levels of p-ERK1/2 and p-AKT were unaltered in the spinal cords with AAV-LRRC4 injection, indicating that the down-regulation of NF-κB p65 was specific (Fig. 7d). These results again suggested that NF-κB serves as a key downstream signaling molecule to mediate the function of LRRC4 in protecting mice from CNS autoimmunity.

## Discussion

LRRC4, which was first cloned by our laboratory, is mainly expressed in the central nervous system (Zhang et al. 2005). Our previous studies showed that LRRC4 regulates cytokine-induced NF-κB activation in glioma cells and that LRRC4 regulates the ERK/MAPK and the PI3K/AKT signaling pathway and therefore modulates cell proliferation, migration, and invasion (Li et al. 2014). LRRC4 (NGL-2) serves as the receptor for netrin-G2, and LRRC4 (NGL-2) interacts with netrin-G2 to play a role in synapse formation (Kim et al. 2006). LRRC4 promotes hippocampal neuron development, while knock-down of LRRC4 reduces dendritic spine density in the hippocampal CA1 region (DeNardo et al. 2012). In this study, we found that LRRC4 is mainly expressed in neuron of spinal cords, and is decreased in the spinal cords of the EAE mice. We further constructed the EAE model in WT mice and LRRC4^−/−^ mice and discovered that the degree of disease is aggravated in LRRC4^−/−^ mice accompanied by increased demyelination, neuron damage, enhanced lymphocytes infiltration into the spinal cords, and elevated microglia and astrocyte proliferation and activation. Ectopic expression of LRRC4 by injecting AAV-LRRC4 virus vector alleviates the progression of EAE mice suggests that LRRC4 may play a neuroprotective role in in the pathogenesis of EAE.

EAE is an inflammatory disorder characterized by demyelination of white matter accompanied by neurodegenerative lesions. Changes in synapse have been found in the CNS of EAE mice including the spinal cord, hippocampus, cerebellum, striatum, and cortex (Haider et al. 2016). The inflammatory environment of the CNS may be the main cause of neurological changes and synaptic loss. The oxidative stress, mitochondrial damage and ion channel dysfunction caused by chronic inflammation have a continuous effect on neurons, leading to neuron death (Ellwardt et al. 2014). We speculated that expression of LRRC4 in neurons may protect the neurons from immune cells attack and oxidative stress. In the CNS of EAE mice, the degenerated myelin and oligodendrocytes would then be phagocytosed by activated microglia, leading to antigen presentation and enhanced myelin-specific autoimmune responses. RNA sequencing results from spinal cord showed that LRRC4 deletion mainly affects neuroactive ligand-receptor interaction, dentate gyrus and hippocampus development, learning, memory and cognition, cell adhension, RNA processing and cellular response to interferon-garmma, astrocyte differentiation, and involving in amyotrophic lateral sclerosis, Huntington desease, viral myocarditis.

RNA sequence analysis also showed that Rab7b expression was the elevated gene in mice with LRRC4 deletion involving amyotrophic lateral sclerosis. We have authenticated that Rab7b was increased in spinal cords of EAE mice, and was negative regulated by LRRC4. Rab7b is a member of small GTPase family and regulates transport between various compartments of the endomembrane system in eukaryotic cells (Yang et al. 2004). Earlier findings demonstrated that Rab7b attenuates TLR4 and TLR9 expression and inhibits NF-κB while decreasing the production of TNF-α, IL-6, NO and IFN-β in macrophages (Wang et al. 2007). However, a separate study showed that Rab7b promotes PMA-induced NF-κB activation and IL-6 production in megakaryocytes (He et al. 2011). Rab7b is reportedly up-regulated in the transient middle cerebral artery occlusion (tMCAO) model, while overexpression of Rab7b in the brain can reduce cerebral infarction of tMCAO and improve neurological functions (Qi et al. 2019). In our study, we found that overexpression of LRRC4 suppresses Rab7b expression in neuron and knockdown of Rab7b increases NF-κB p65, suggesting that LRRC4 regulates NF-κB p65 is dependent on Rab7b and Rab7b might serve as a critical downstream target of LRRC4 to exert LRRC4 protective function from EAE development.

We found that NF-κB p65 expression is elevated while the ratio of p-AKT/AKT is reduced in the spinal cords of LRRC4^−/−^ mice, suggesting that NF-κB and PI3K/AKT signaling act as key downstream effectors of LRRC4 in modulating EAE progression. The NF-κB signaling cascade plays a critical role in the regulation of immune and inflammatory responses and the function of resident cells of the CNS that are implicated in the pathogenesis of MS and EAE (Camandola et al. 2007). NF-κB is activated in the CNS of EAE and persists throughout the development of the disease (Kaltschmidt et al. 1994). Upon activation, NF-κB induces the expression of inflammatory factors and triggers the immune responses during EAE progression (Hwang et al. 2011). Meanwhile, the PI3K/AKT signaling pathway in oligodendrocytes has a critical function in the myelination process after demyelinating injury in EAE (Flores et al. 2008). Our findings suggest the following model: LRRC4 deletion causes up-regulation of NF-κB p65 and down-regulation of p-AKT/AKT, leading to altered secretion of inflammatory factors such as IL-6, IFN-γ, IL-10, TGF-β and TNF-α. The level of IL-6 is elevated in the central nervous system of MS patients and EAE mice, while IL-6 inhibitors can inhibit the differentiation of Th1 and Th17 cells and reduce the pathogenesis of EAE (Serada et al. 2008). IL-6 also inhibits the function of Treg cells and regulates the balance of Treg/Th17 cells (Kimura et al. 2010). In addition, IFN-γ also plays a role in the pathogenesis of MS and EAE (Popko et al. 1997). Specifically, IFN-γ is elevated in the serum of MS patients, while administration of IFN-γ to MS patients in a clinical trial aggravates the development of the disease (Arellano et al. 2015). In addition, IFN-γ promotes development of Th1 cells but inhibits Th17 cell differentiation from naïve precursor cells (Kalinke et al. 2012). Thus, these earlier findings support our notion that the increase in Th1 cells and the decrease in Treg cells in EAE mice with LRRC4 deletion may be attributed to elevated levels of IL-6 and IFN-γ.

Our model is also in agreement with the previously established role of TGF-β, IL-10 and TNF-α, all of which are down-regulated in the EAE mice with LRRC4 deletion. TGF-β reportedly plays a role in regulating T cell differentiation and function (Lee et al. 2017). For instance, TGF-β in combination with IL-2 and retinoic acid can induce differentiation of primary CD4^+^ cells into Treg cells, while TGF-β in combination with IL-6 can promote the differentiation of Th17 cells (Chen et al. 2003; Das et al. 2009). In contrast, TGF-β functions as an inhibitor of Th1 cell differentiation (Lin et al. 2005). In addition, myelin immune-reactive T cells stimulated by TGF-β are unable to differentiate into effector T cells and cannot induce EAE (Veldhoen et al. 2006). IL-10 is an anti-inflammatory cytokine and can inhibit inflammation in autoimmune diseases. IL-10-deficient mice develop more severe EAE, indicating that IL-10 has a protective role in the pathogenesis of EAE (Murai et al. 2009). The immunosuppressive function of IL-10 involves the regulation of antigen-presenting cells (APCs), inhibition of T cell proliferation, and maintenance of Treg cell function (Rynda-Apple et al. 2011). As such, the reduction of TGF-β and IL-10 levels in EAE mice with LRRC4 deletion, as shown in our present study, may inhibit Treg cell differentiation and function. It has been shown previously that the level of TNF-α is elevated in the CNS of MS patients and EAE models (Rossi et al. 2014). Antibodies against TNF-α or TNFRl can inhibit the development of EAE (Kaltsonoudis et al. 2014). Deletion of TNF-α in mice delays the onset of EAE without changing in the incidence and severity of EAE (Kassiotis et al. 2001). However, TNFR2 deletion in mice causes an increase in inflammatory responses, demyelination, and the severity of EAE. Thus, TNFR2 promotes the function of oligodendrocytes, inhibits lymphocyte infiltration, and plays a protective role in EAE (Kollias et al. 1999). As such, the reduced TNF-α expression we have observed in EAE mice with LRRC4 deletion may explain the elevated lymphocyte infiltration.

## Conclusions

**Fig. 8:**
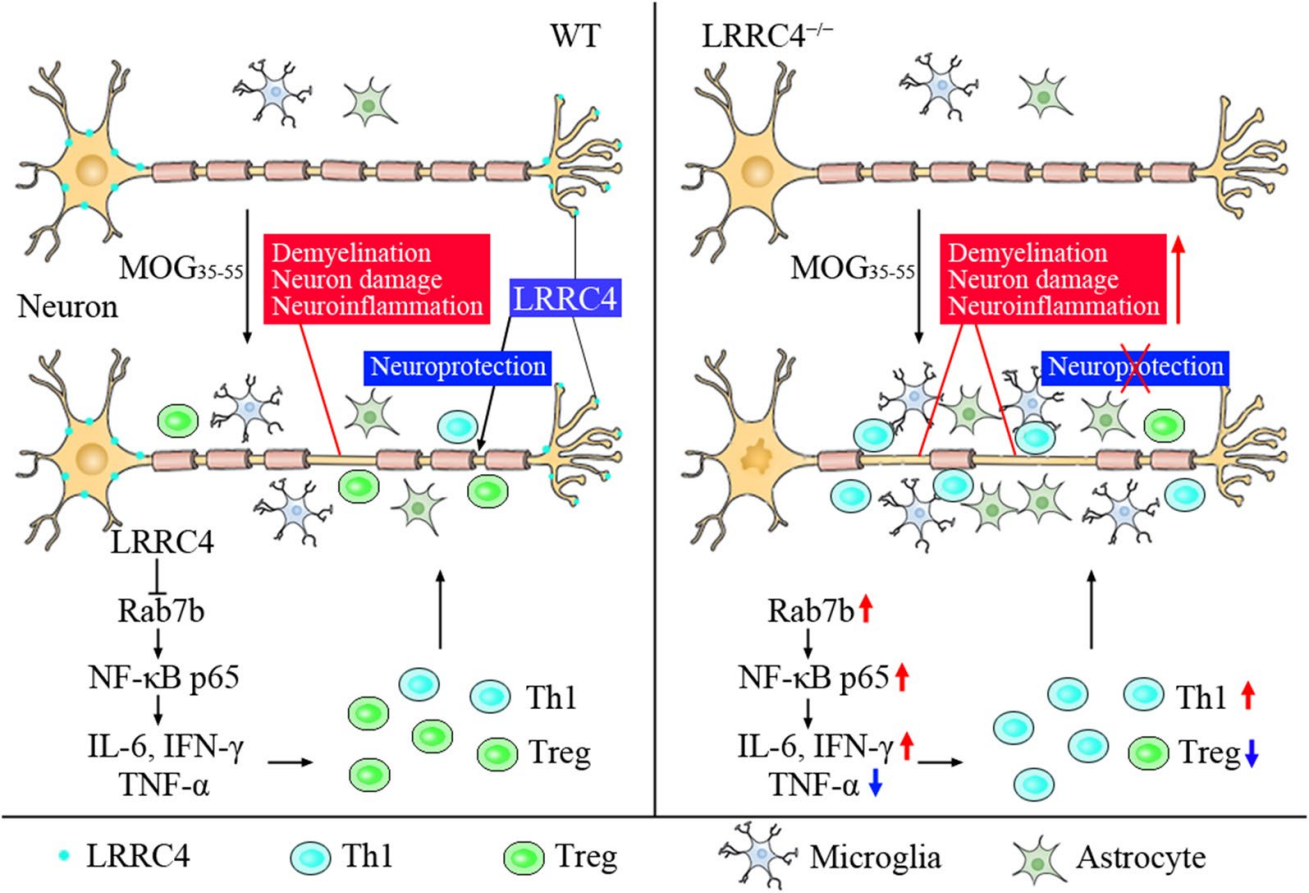
Schematic diagram of LRRC4 play a neuroprotective role in EAE. LRRC4 inhibited NF-κB p65 expression by down-regulating Rab7b. Deletion of LRRC4 accelerated demyelination, neuron damage, neuroinflammation, and Th1 immune response in EAE mice

In summary, our experiments demonstrate a critical role of LRRC4 in EAE progression (Fig. 8). LRRC4 decrease in spinal cords of EAE mice. LRRC4 expresses mainly in neurons and palys a meuroprotective role in EAE mice for that deletion of LRRC4 aggravates demyelination, neuron damage and neuroinflammation. LRRC4 deficiency increases NF-κB p65 and decreased p-AKT, leads to up-regulation of IFN-γ and IL-6, and down-regulation of IL-10, TNF-α and TGF-β, results in increased Th1 cells and decreased Treg cells. LRRC4 regulates Rab7b expression may involve in the regulation of NF-κB and pathology of EAE. Restoring LRRC4 represents a new strategy for preventing exacerbations and therapy in EAE.

## Supplementary Information

**Additional file 1: Fig. S1.** EAE mice were induced by MOG35-55. a Clinical scores of naïve and MOG-immunized mice. Data represent the mean clinical scores of 10 mice per group ± SEM. b Histopathological analysis of neuroinflammation and demyelination in spinal cords of naïve and EAE mice. Sections were stained by the use of H&E staining and LFP staining. **Fig. S2.** The auditory brainstem response (ABR) of LRRC4^−/−^ mice compared with that of the WT control. a Schemeatic diagrams showing construction of LRRC4^−/−^ mice. b Real-time PCR analysis of LRRC4 mRNA levels in brains and spinal cords of WT or LRRC4^−/−^ mice. c Western blotting analysis of LRRC4 protein levels in brains and spinal cords of WT or LRRC4^−/−^ mice. d Representative diagrams of ABR of WT or LRRC4^−/−^ mice showing waves at different decibel levels of click stimuli. e The threshold of ABR of WT or LRRC4^−/−^ mice. Results are shown as means ± SEM (n=8). **p < 0.01. **Fig. S3.** Analysis of differentially expressed genes between EAE-WT and EAE-LRRC4^−/−^ mice by RNA-seq. a A heatmap showing DEGs in spinal cord of EAE-WT versus EAE-LRRC4^−/−^ mice. b The top 10 GO terms of up-regulated and down-regulated DEGs between EAE-WT and EAE-LRRC4^−/−^ mice. c The top 10 KEGG pathways of up-regulated and down-regulated DEGs between EAE-WT and EAE-LRRC4^−/−^ mice. d The comparison of GO terms between up-regulated DEGs and down-regulated DEGs. e Comparison of KEGG pathways between up-regulated DEGs and down-regulated DEGs.

## Data Availability

The data used in this article are available to researchers subject to confidentiality if necessary.

## Abbreviations

LRRC4: Leucine rich repeat containing 4
NGL-2: Netrin-G ligand-2
ASD: Autism spectrum disorder
MS: Multiple sclerosis
CNS: Central nervous system
EAE: Experimental autoimmune encephalomyelitis
MOG: Oligodendrocyte glycoprotein peptide
LFB: Luxol fast blue
AAV: Adeno-associated virus
TNF: Tumor necrosis factor
ROR-γ: Tretinoid-related orphan receptor-γt
GM-CSF: Granulocyte–macrophage colony-stimulating factor
MBP: Myelin basic protein
NMDARs: N-methyl-D-aspartate receptors
PDPK1: Phosphoinositide-dependent protein kinase 1
ABR: Auditory brainstem response
