# Supplementary material for: LRRC4 functions as a neuron-protective role in experimental autoimmune encephalomyelitis
Source: Mol Med. 2021 May 1;27:44. doi: 10.1186/s10020-021-00304-4 (PMC8088686; doi:10.1186/s10020-021-00304-4)
Supplement: Supplementary file 1 — Additional file 1: Fig. S1. EAE mice were induced by MOG35-55. a Clinical scores of naïve and MOG-immunized mice. Data represent the mean clinical scores of 10 mice per group ± SEM. b Histopathological analysis of neuroinflammation and demyelination in spinal cords of naïve and EAE mice. Sections were stained by the use of H&E staining and LFP staining. Fig. S2. The auditory brainstem response (ABR) of LRRC4−/− mice compared with that of the WT control. a Schemeatic diagrams showing construction of LRRC4−/− mice. b Real-time PCR analysis of LRRC4 mRNA levels in brains and spinal cords of WT or LRRC4−/− mice. c Western blotting analysis of LRRC4 protein levels in brains and spinal cords of WT or LRRC4−/− mice. d Representative diagrams of ABR of WT or LRRC4−/− mice showing waves at different decibel levels of click stimuli. e The threshold of ABR of WT or LRRC4−/− mice. Results are shown as means ± SEM (n=8). **p < 0.01. Fig. S3. Analysis of differentially expressed genes between EAE-WT and EAE-LRRC4−/− mice by RNA-seq. a A heatmap showing DEGs in spinal cord of EAE-WT versus EAE-LRRC4−/− mice. b The top 10 GO terms of up-regulated and down-regulated DEGs between EAE-WT and EAE-LRRC4−/− mice. c The top 10 KEGG pathways of up-regulated and down-regulated DEGs between EAE-WT and EAE-LRRC4−/− mice. d The comparison of GO terms between up-regulated DEGs and down-regulated DEGs. e Comparison of KEGG pathways between up-regulated DEGs and down-regulated DEGs. [file 10020_2021_304_MOESM1_ESM.docx]

**
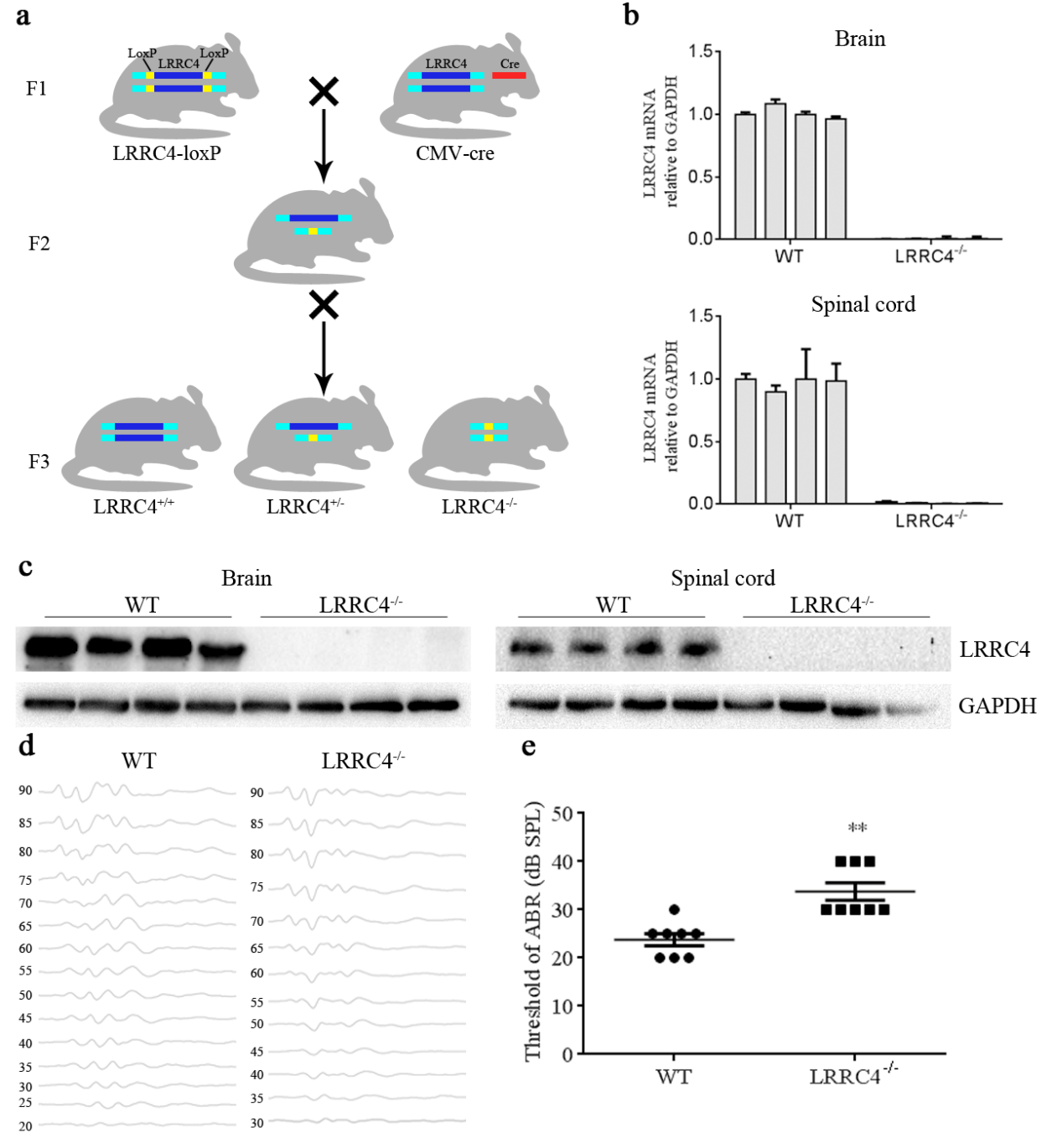
** Supplementary Fig. 1 The auditory brainstem response (ABR) of LRRC4^-/-^ mice compared with that of the WT control. **a** Schemeatic diagrams showing construction of LRRC4^-/-^ mice. **b** Real-time PCR analysis of LRRC4 mRNA levels in brains and spinal cords of WT or LRRC4^-/-^ mice. **c** Western blotting analysis of LRRC4 protein levels in brains and spinal cords of WT or LRRC4^-/-^ mice. **d** Representative diagrams of ABR of WT or LRRC4^-/-^ mice showing waves at different decibel levels of click stimuli. **e** The threshold of ABR of WT or LRRC4^-/-^ mice. Results are shown as means ± SEM (n=8). ***p* < 0.01.


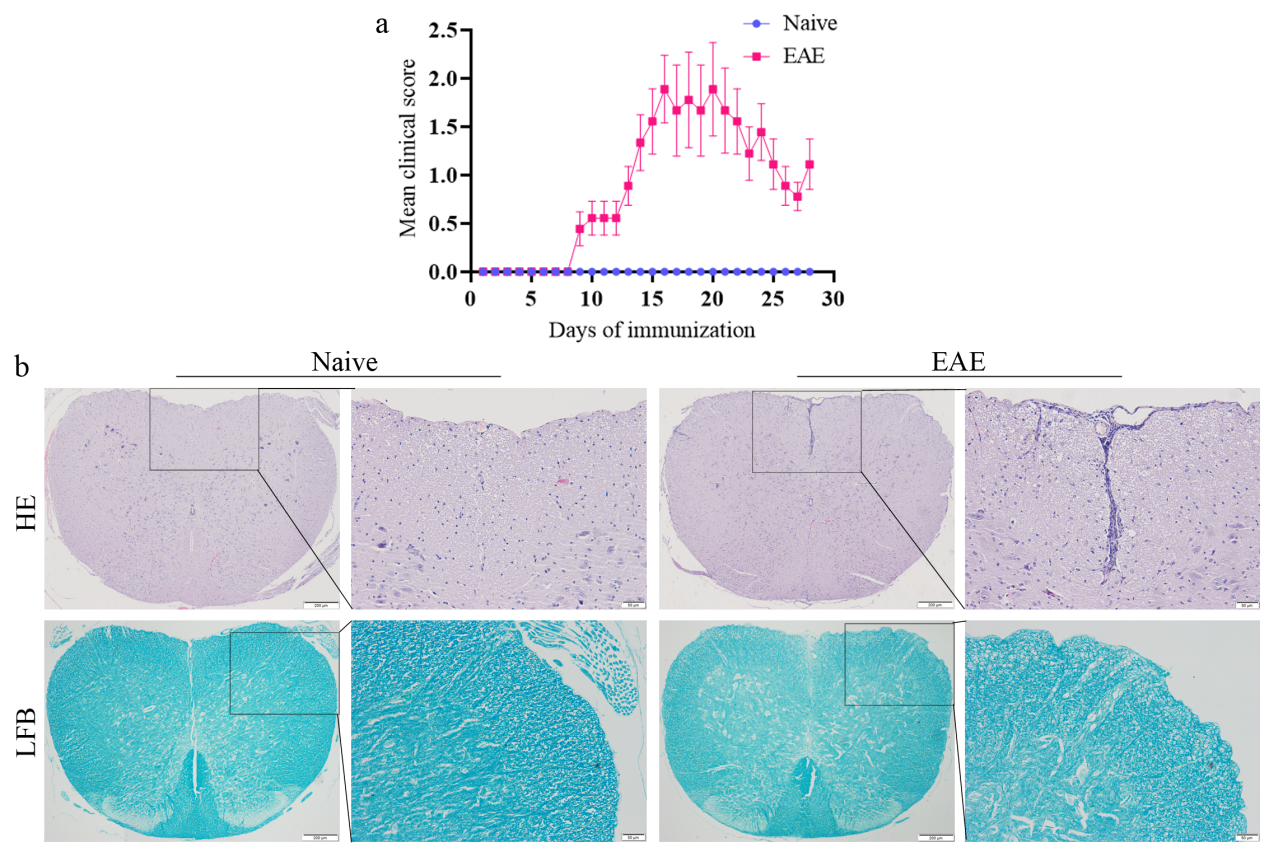


Supplementary Fig. 2 EAE mice were induced by MOG_35-55_. **a** Clinical scores of naive and MOG-immunized mice. Data represent the mean clinical scores of 10 mice per group ± SEM. **b** Histopathological analysis of neuroinflammation and demyelination in spinal cords of naive and EAE mice. Sections were stained by the use of H&E staining and LFP staining.


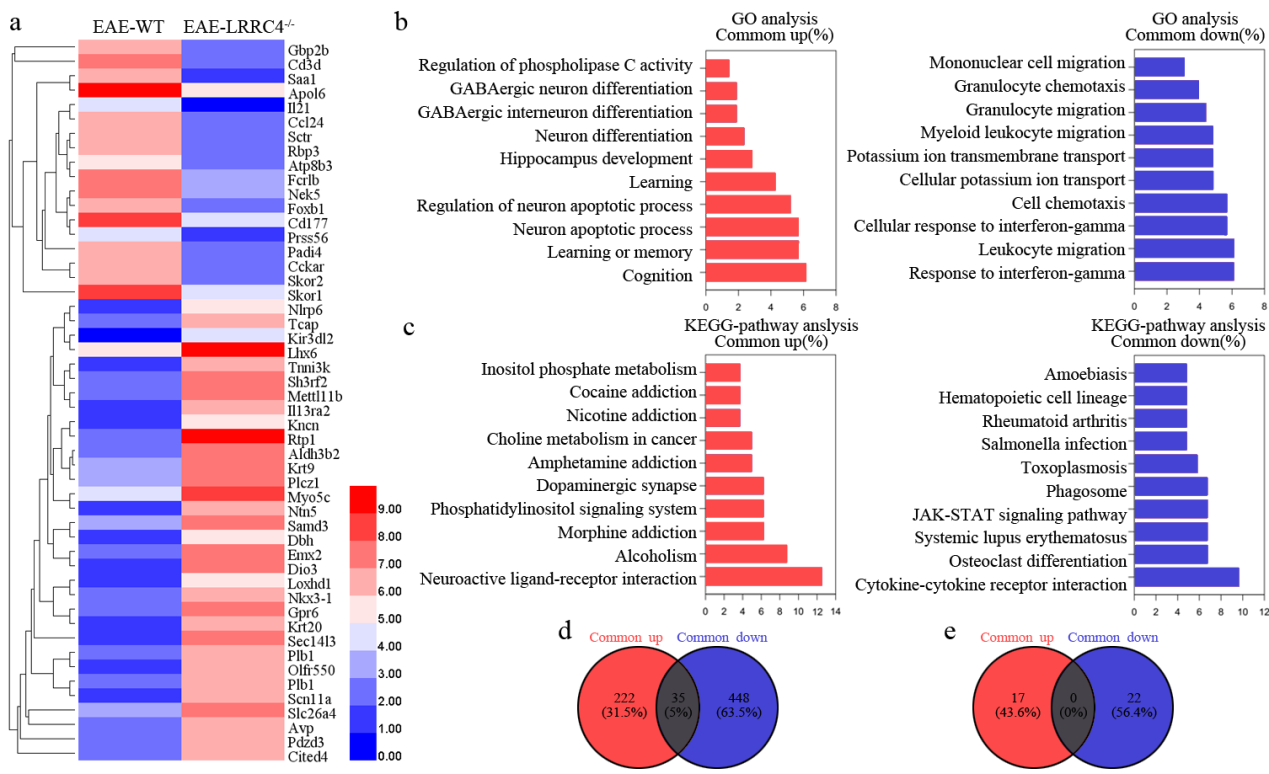


Supplementary Fig. 3 Analysis of differentially expressed genes between EAE-WT and EAE-LRRC4^-/-^ mice by RNA seq. **a** A heatmap showing DEGs in spinal cord of EAE-WT versus EAE-LRRC4^-/-^ mice. **b** The top 10 GO terms of up-regulated and down-regulated DEGs between EAE-WT and EAE-LRRC4^-/-^ mice. **c** The top 10 KEGG pathways of up-regulated and down-regulated DEGs between EAE-WT and EAE-LRRC4^-/-^ mice. **d** The comparison of GO terms between up-regulated DEGs and down-regulated DEGs. **e** Comparison of KEGG pathways between up-regulated DEGs and down-regulated DEGs.
